# Meranzin hydrate from *Muraya paniculata*

**DOI:** 10.1107/S1600536810005386

**Published:** 2010-02-13

**Authors:** Euis Julaeha, Unang Supratman, Mat Ropi Mukhtar, Khalijah Awang, Seik Weng Ng

**Affiliations:** a Department of Chemistry, Faculty of Mathematics and Natural Sciences, Padjadjaran University, Jatinangor 45363, West Java, Indonesia; b Department of Chemistry, University of Malaya, 50603 Kuala Lumpur, Malaysia

## Abstract

The coumarin ring system in the title compound, C_15_H_18_O_5_ [IUPAC name: 8-(2,3-dihydr­oxy-3-methyl­butyl)-7-meth­oxy-2*H*-1-benzopyran-2-one], isolated from *Muraya paniculata*, is planar (r.m.s. deviation 0.017 Å). In the crystal, the two hydr­oxy groups are involved in O—H⋯O hydrogen bonding with adjacent mol­ecules, forming a sheet structure.

## Related literature

For the asymmetric synthesis and absolute configuration of meranzin hydrate, see: Grundon & McColl (1975 ▶).
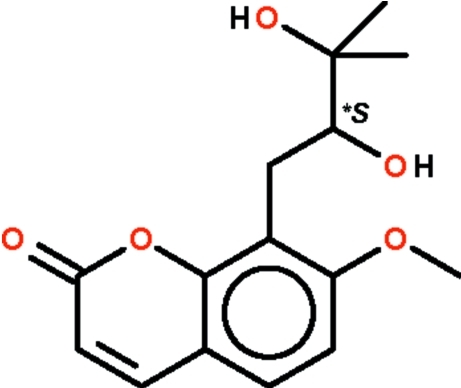


## Experimental

### 

#### Crystal data

C_15_H_18_O_5_
*M* *_r_* = 278.29
Monoclinic, 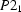

*a* = 5.8061 (7) Å
*b* = 10.5146 (13) Å
*c* = 11.4477 (14) Å
β = 91.547 (2)°
*V* = 698.61 (15) Å^3^
*Z* = 2
Mo *K*α radiation
μ = 0.10 mm^−1^
*T* = 293 K
0.35 × 0.15 × 0.15 mm

#### Data collection

Bruker SMART APEX diffractometer
6694 measured reflections
1699 independent reflections
1338 reflections with *I* > 2σ(*I*)
*R*_int_ = 0.040

#### Refinement

*R*[*F* ^2^ > 2σ(*F* ^2^)] = 0.038
*wR*(*F* ^2^) = 0.102
*S* = 1.00
1699 reflections
192 parameters
3 restraints
H atoms treated by a mixture of independent and constrained refinement
Δρ_max_ = 0.12 e Å^−3^
Δρ_min_ = −0.16 e Å^−3^

### 

Data collection: *APEX2* (Bruker, 2008 ▶); cell refinement: *SAINT* (Bruker, 2008 ▶); data reduction: *SAINT*; program(s) used to solve structure: *SHELXS97* (Sheldrick, 2008 ▶); program(s) used to refine structure: *SHELXL97* (Sheldrick, 2008 ▶); molecular graphics: *X-SEED* (Barbour, 2001 ▶); software used to prepare material for publication: *publCIF* (Westrip, 2010 ▶).

## Supplementary Material

Crystal structure: contains datablocks global, I. DOI: 10.1107/S1600536810005386/bt5194sup1.cif

Structure factors: contains datablocks I. DOI: 10.1107/S1600536810005386/bt5194Isup2.hkl

Additional supplementary materials:  crystallographic information; 3D view; checkCIF report

## Figures and Tables

**Table 1:** Hydrogen-bond geometry (Å, °)

*D*—H⋯*A*	*D*—H	H⋯*A*	*D*⋯*A*	*D*—H⋯*A*
O4—H4⋯O2^i^	0.84 (1)	2.01 (1)	2.842 (3)	169 (5)
O5—H5⋯O2^ii^	0.85 (1)	2.12 (2)	2.936 (3)	163 (4)

Symmetry codes: (i) 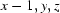; (ii) 
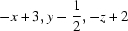
.

**Fig. 1.**
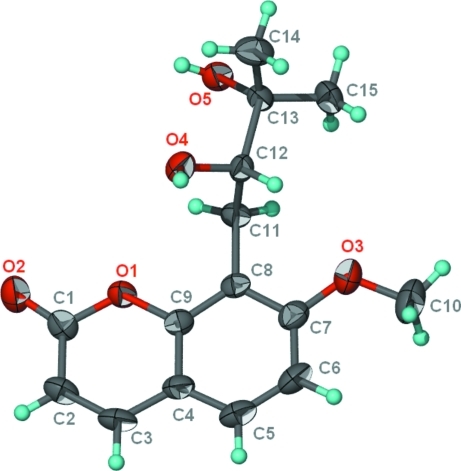
Anisotropic displacement ellipsoid plot (Barbour, 2001) of C15H18O5; at the 50% probability level; hydrogen atoms are drawn as spheres of arbitrary radius.

## supplementary crystallographic information

### Comment

*Muraya paniculata* (Rutaceae, known as kemuning in Indonesia) is a perennial herb having succulent leaves. The plant is used for the treatment of orchitis, bronchitis and urine infections.

### Experimental

*M. paniculata* was collected in from Bandung, Indonesia. The plant was identified by the Department of Biology of Padjadjaran University. The dried leaves of *M. paniculata* (4 kg) was extracted exhaustively by methanol at room temperature and then concentrated to yield a methanol extract (438 g); 200 g was partitioned between *n*-hexane and methanol containing 10% water. The aqueous extract was extracted with ethyl acetate. The ethyl acetate portion was removed and subjected to column chromatography on silica gel 60 by using a step gradient of *n*-hexane–ethyl acetate–methanol. The fraction eluted by *n*-hexane/ethyl acetate (1:4) was further separated by column chromatography on silica gel (chloroform:ethyl acetate 1:1) to give meranzin hydrate, 8-[2,3-dihydroxy-3-methylbutyl]-7-methoxy-2*H*-1-benzopyran-2-one (12 mg).

### Refinement

Carbon-bound H atoms were placed in calculated positions (C—H 0.93 to 0.97 Å) and were included in the refinement in the riding model approximation, with *U*(H) set to 1.2 to 1.5*U*(C).

The oxygen-bound H atoms were located in a difference Fourier map, and were refined isotropically with a distance restraint of O—H 0.84 (1) Å.

In the absence of anomalous scatterers, Friedel pairs were merged. The absolute configuration was set to match the one determined by the asymmetric synthesis of meranzin (Grundon & McColl, 1975).

### Crystal data

**Table:**

C_15_H_18_O_5_	*F*(000) = 296
*M**_r_* = 278.29	*D*_x_ = 1.323 Mg m^−^^3^
Monoclinic, *P*2_1_	Mo *K*α radiation, λ = 0.71073 Å
Hall symbol: P 2yb	Cell parameters from 1731 reflections
*a* = 5.8061 (7) Å	θ = 2.6–22.3°
*b* = 10.5146 (13) Å	µ = 0.10 mm^−^^1^
*c* = 11.4477 (14) Å	*T* = 293 K
β = 91.547 (2)°	Prism, colourless
*V* = 698.61 (15) Å^3^	0.35 × 0.15 × 0.15 mm
*Z* = 2	

### Data collection

**Table:**

Bruker SMART APEX diffractometer	1338 reflections with *I* > 2σ(*I*)
Radiation source: fine-focus sealed tube	*R*_int_ = 0.040
graphite	θ_max_ = 27.5°, θ_min_ = 1.8°
ω scans	*h* = −7→7
6694 measured reflections	*k* = −11→13
1699 independent reflections	*l* = −14→14

### Refinement

**Table:**

Refinement on *F*^2^	Primary atom site location: structure-invariant direct methods
Least-squares matrix: full	Secondary atom site location: difference Fourier map
*R*[*F*^2^ > 2σ(*F*^2^)] = 0.038	Hydrogen site location: inferred from neighbouring sites
*wR*(*F*^2^) = 0.102	H atoms treated by a mixture of independent and constrained refinement
*S* = 1.00	*w* = 1/[σ^2^(*F*_o_^2^) + (0.0603*P*)^2^] where *P* = (*F*_o_^2^ + 2*F*_c_^2^)/3
1699 reflections	(Δ/σ)_max_ = 0.001
192 parameters	Δρ_max_ = 0.12 e Å^−^^3^
3 restraints	Δρ_min_ = −0.16 e Å^−^^3^

### Fractional atomic coordinates and isotropic or equivalent isotropic displacement parameters (Å^2^)

**Table:**

	*x*	*y*	*z*	*U*_iso_*/*U*_eq_	
O1	1.4383 (3)	0.50000 (16)	0.83522 (15)	0.0436 (4)	
O2	1.7272 (3)	0.51296 (19)	0.96204 (18)	0.0592 (5)	
O3	0.8098 (4)	0.4410 (2)	0.57958 (18)	0.0677 (6)	
O4	1.0213 (3)	0.30444 (18)	0.91374 (15)	0.0500 (5)	
O5	1.1435 (3)	0.06759 (16)	0.79342 (17)	0.0490 (4)	
C1	1.5866 (4)	0.5712 (3)	0.9034 (2)	0.0461 (6)	
C2	1.5618 (5)	0.7069 (3)	0.8999 (3)	0.0553 (7)	
H2	1.6632	0.7576	0.9436	0.066*	
C3	1.3954 (5)	0.7612 (3)	0.8350 (3)	0.0567 (7)	
H3	1.3813	0.8493	0.8345	0.068*	
C4	1.2374 (5)	0.6858 (2)	0.7657 (2)	0.0476 (6)	
C5	1.0585 (5)	0.7350 (3)	0.6972 (3)	0.0596 (8)	
H5A	1.0373	0.8226	0.6939	0.071*	
C6	0.9124 (5)	0.6572 (3)	0.6344 (3)	0.0610 (8)	
H6	0.7931	0.6918	0.5888	0.073*	
C7	0.9433 (5)	0.5268 (3)	0.6391 (2)	0.0513 (7)	
C8	1.1185 (4)	0.4708 (2)	0.7080 (2)	0.0415 (5)	
C9	1.2625 (4)	0.5534 (2)	0.7693 (2)	0.0400 (5)	
C10	0.6251 (5)	0.4863 (4)	0.5056 (3)	0.0792 (11)	
H10A	0.5429	0.4153	0.4723	0.119*	
H10B	0.6864	0.5372	0.4442	0.119*	
H10C	0.5222	0.5367	0.5507	0.119*	
C11	1.1382 (4)	0.3280 (2)	0.7160 (2)	0.0419 (5)	
H11A	1.2906	0.3052	0.7459	0.050*	
H11B	1.1181	0.2912	0.6387	0.050*	
C12	0.9563 (4)	0.2742 (2)	0.79647 (19)	0.0388 (5)	
H12	0.8090	0.3159	0.7777	0.047*	
C13	0.9231 (4)	0.1295 (2)	0.7838 (2)	0.0406 (5)	
C14	0.7685 (5)	0.0804 (3)	0.8780 (3)	0.0628 (8)	
H14A	0.7520	−0.0100	0.8704	0.094*	
H14B	0.6199	0.1200	0.8700	0.094*	
H14C	0.8354	0.1002	0.9534	0.094*	
C15	0.8225 (5)	0.0987 (3)	0.6642 (3)	0.0628 (8)	
H15A	0.7866	0.0096	0.6600	0.094*	
H15B	0.9324	0.1193	0.6059	0.094*	
H15C	0.6846	0.1474	0.6505	0.094*	
H4	0.935 (6)	0.363 (3)	0.937 (4)	0.119 (17)*	
H5	1.183 (6)	0.068 (4)	0.8650 (12)	0.089 (12)*	

### Atomic displacement parameters (Å^2^)

**Table:**

	*U*^11^	*U*^22^	*U*^33^	*U*^12^	*U*^13^	*U*^23^
O1	0.0453 (9)	0.0336 (9)	0.0515 (10)	0.0040 (7)	−0.0063 (7)	−0.0039 (7)
O2	0.0526 (10)	0.0556 (12)	0.0683 (12)	0.0103 (9)	−0.0159 (9)	−0.0154 (10)
O3	0.0719 (13)	0.0715 (15)	0.0581 (12)	−0.0035 (11)	−0.0275 (10)	0.0085 (11)
O4	0.0630 (11)	0.0466 (11)	0.0400 (9)	0.0096 (9)	−0.0081 (8)	−0.0065 (8)
O5	0.0460 (9)	0.0389 (10)	0.0616 (12)	0.0071 (7)	−0.0058 (8)	−0.0042 (9)
C1	0.0428 (12)	0.0441 (15)	0.0512 (14)	0.0015 (11)	−0.0013 (11)	−0.0098 (12)
C2	0.0581 (15)	0.0403 (15)	0.0676 (18)	−0.0087 (12)	−0.0002 (14)	−0.0088 (13)
C3	0.0722 (17)	0.0299 (13)	0.0683 (18)	−0.0035 (13)	0.0078 (15)	−0.0034 (12)
C4	0.0567 (14)	0.0356 (14)	0.0508 (15)	0.0018 (12)	0.0047 (12)	0.0054 (12)
C5	0.0716 (18)	0.0427 (16)	0.0645 (18)	0.0122 (14)	0.0013 (15)	0.0140 (14)
C6	0.0656 (17)	0.0572 (19)	0.0599 (17)	0.0137 (14)	−0.0073 (14)	0.0186 (15)
C7	0.0573 (15)	0.0544 (17)	0.0416 (14)	0.0014 (13)	−0.0065 (12)	0.0098 (13)
C8	0.0475 (12)	0.0371 (12)	0.0398 (13)	0.0011 (10)	−0.0002 (10)	0.0037 (10)
C9	0.0450 (12)	0.0345 (13)	0.0406 (13)	0.0035 (10)	0.0023 (10)	0.0042 (10)
C10	0.0586 (16)	0.114 (3)	0.0636 (19)	0.001 (2)	−0.0203 (15)	0.016 (2)
C11	0.0447 (12)	0.0353 (12)	0.0455 (13)	−0.0001 (10)	−0.0016 (10)	−0.0042 (11)
C12	0.0402 (10)	0.0373 (12)	0.0384 (12)	0.0051 (10)	−0.0072 (9)	−0.0023 (10)
C13	0.0373 (10)	0.0343 (12)	0.0499 (13)	−0.0004 (10)	−0.0055 (9)	−0.0011 (11)
C14	0.0524 (15)	0.0546 (17)	0.082 (2)	−0.0116 (13)	0.0071 (14)	0.0101 (16)
C15	0.0668 (17)	0.0530 (17)	0.0672 (18)	−0.0063 (14)	−0.0239 (14)	−0.0130 (15)

### Geometric parameters (Å, °)

**Table:**

O1—C1	1.369 (3)	C7—C8	1.400 (3)
O1—C9	1.373 (3)	C8—C9	1.383 (3)
O2—C1	1.209 (3)	C8—C11	1.509 (3)
O3—C7	1.361 (3)	C10—H10A	0.9600
O3—C10	1.430 (3)	C10—H10B	0.9600
O4—C12	1.421 (3)	C10—H10C	0.9600
O4—H4	0.840 (10)	C11—C12	1.529 (3)
O5—C13	1.437 (3)	C11—H11A	0.9700
O5—H5	0.846 (10)	C11—H11B	0.9700
C1—C2	1.434 (4)	C12—C13	1.540 (3)
C2—C3	1.332 (4)	C12—H12	0.9800
C2—H2	0.9300	C13—C15	1.510 (4)
C3—C4	1.434 (4)	C13—C14	1.512 (4)
C3—H3	0.9300	C14—H14A	0.9600
C4—C5	1.384 (4)	C14—H14B	0.9600
C4—C9	1.401 (3)	C14—H14C	0.9600
C5—C6	1.369 (4)	C15—H15A	0.9600
C5—H5A	0.9300	C15—H15B	0.9600
C6—C7	1.383 (4)	C15—H15C	0.9600
C6—H6	0.9300		
			
C1—O1—C9	122.44 (19)	O3—C10—H10C	109.5
C7—O3—C10	118.9 (3)	H10A—C10—H10C	109.5
C12—O4—H4	109 (3)	H10B—C10—H10C	109.5
C13—O5—H5	107 (3)	C8—C11—C12	110.6 (2)
O2—C1—O1	116.4 (2)	C8—C11—H11A	109.5
O2—C1—C2	125.8 (3)	C12—C11—H11A	109.5
O1—C1—C2	117.9 (2)	C8—C11—H11B	109.5
C3—C2—C1	120.8 (3)	C12—C11—H11B	109.5
C3—C2—H2	119.6	H11A—C11—H11B	108.1
C1—C2—H2	119.6	O4—C12—C11	108.45 (18)
C2—C3—C4	121.0 (2)	O4—C12—C13	109.84 (19)
C2—C3—H3	119.5	C11—C12—C13	113.30 (19)
C4—C3—H3	119.5	O4—C12—H12	108.4
C5—C4—C9	117.6 (3)	C11—C12—H12	108.4
C5—C4—C3	124.4 (3)	C13—C12—H12	108.4
C9—C4—C3	118.0 (2)	O5—C13—C15	107.1 (2)
C6—C5—C4	121.3 (3)	O5—C13—C14	109.6 (2)
C6—C5—H5A	119.3	C15—C13—C14	110.5 (2)
C4—C5—H5A	119.3	O5—C13—C12	109.36 (17)
C5—C6—C7	119.6 (3)	C15—C13—C12	110.0 (2)
C5—C6—H6	120.2	C14—C13—C12	110.2 (2)
C7—C6—H6	120.2	C13—C14—H14A	109.5
O3—C7—C6	124.5 (2)	C13—C14—H14B	109.5
O3—C7—C8	113.5 (2)	H14A—C14—H14B	109.5
C6—C7—C8	122.0 (3)	C13—C14—H14C	109.5
C9—C8—C7	116.2 (2)	H14A—C14—H14C	109.5
C9—C8—C11	123.4 (2)	H14B—C14—H14C	109.5
C7—C8—C11	120.4 (2)	C13—C15—H15A	109.5
O1—C9—C8	116.90 (19)	C13—C15—H15B	109.5
O1—C9—C4	119.8 (2)	H15A—C15—H15B	109.5
C8—C9—C4	123.3 (2)	C13—C15—H15C	109.5
O3—C10—H10A	109.5	H15A—C15—H15C	109.5
O3—C10—H10B	109.5	H15B—C15—H15C	109.5
H10A—C10—H10B	109.5		
			
C9—O1—C1—O2	176.4 (2)	C1—O1—C9—C4	3.2 (3)
C9—O1—C1—C2	−3.2 (4)	C7—C8—C9—O1	−178.6 (2)
O2—C1—C2—C3	−177.7 (3)	C11—C8—C9—O1	3.6 (3)
O1—C1—C2—C3	1.9 (4)	C7—C8—C9—C4	0.8 (4)
C1—C2—C3—C4	−0.6 (5)	C11—C8—C9—C4	−177.1 (3)
C2—C3—C4—C5	179.2 (3)	C5—C4—C9—O1	179.5 (2)
C2—C3—C4—C9	0.5 (4)	C3—C4—C9—O1	−1.8 (4)
C9—C4—C5—C6	−0.6 (4)	C5—C4—C9—C8	0.1 (4)
C3—C4—C5—C6	−179.2 (3)	C3—C4—C9—C8	178.9 (2)
C4—C5—C6—C7	0.0 (5)	C9—C8—C11—C12	101.1 (3)
C10—O3—C7—C6	0.6 (4)	C7—C8—C11—C12	−76.7 (3)
C10—O3—C7—C8	−179.7 (2)	C8—C11—C12—O4	−73.0 (2)
C5—C6—C7—O3	−179.4 (3)	C8—C11—C12—C13	164.76 (19)
C5—C6—C7—C8	1.0 (5)	O4—C12—C13—O5	−70.5 (2)
O3—C7—C8—C9	179.0 (2)	C11—C12—C13—O5	51.0 (2)
C6—C7—C8—C9	−1.3 (4)	O4—C12—C13—C15	172.2 (2)
O3—C7—C8—C11	−3.1 (4)	C11—C12—C13—C15	−66.4 (3)
C6—C7—C8—C11	176.6 (3)	O4—C12—C13—C14	50.0 (2)
C1—O1—C9—C8	−177.4 (2)	C11—C12—C13—C14	171.5 (2)

### Hydrogen-bond geometry (Å, °)

**Table:**

*D*—H···*A*	*D*—H	H···*A*	*D*···*A*	*D*—H···*A*
O4—H4···O2^i^	0.84 (1)	2.01 (1)	2.842 (3)	169 (5)
O5—H5···O2^ii^	0.85 (1)	2.12 (2)	2.936 (3)	163 (4)

Symmetry codes: (i) *x*−1, *y*, *z*; (ii) −*x*+3, *y*−1/2, −*z*+2.
